# Pain control and related factors in hospitalized patients

**DOI:** 10.1097/MD.0000000000026768

**Published:** 2021-07-30

**Authors:** Li-Ying Lin, Tzu-Ching Hung, Yen-Ho Lai

**Affiliations:** a Department of Nursing, Kaohsiung Veterans General Hospital, Kaohsiung City, Taiwan; b Department of Nursing, Meiho University, Pingtung County, Taiwan; c National Kaohsiung University of Science and Technology, Kaohsiung City, Taiwan.

**Keywords:** emotional distress, hospitalized patients, pain, related factors

## Abstract

Pain is a common health problem for hospitalized patients. It is necessary to understand the factors that affect patients’ pain to provide individual and complete pain management. This study explored the severity and incidence of pain in hospitalized patients on the admission day, during the hospitalization, and the discharge day, and explored the predictive factors that affect the patient's pain on the discharge day.

This was a retrospective study that reviewed the medical records of 73,814 hospitalized patients from January 2016 to April 2018. The number of pain assessments was 471,339.

The incidence of pain on the discharge day of patients was significantly higher than that on the admission day. The factors that affect and predict the pain of patients on discharge day include the degree of pain on the day of admission, emotional distress on the day of discharge, disease category, gender, age, and length of stay in hospital. It shows that the higher the degree of pain on the day of admission, the higher the degree of emotional distress on the day of discharge, female patients, younger patients, longer hospitalization days, and surgical and gynecological patients have significantly higher pain levels on the day of discharge (*P* < .05).

This study found that the incidence of pain on the discharge day of patients was 46.5%. Previous pain level, disease category, emotional distress, gender, age, and length of hospital stay were important factors affecting patient pain on the discharge day. The influencing factors of patient pain should be fully assessed to provide individual and complete pain management, and improve patient quality of life after discharge.

## 1 Introduction

Pain is an unpleasant sensory and emotional experience associated with, or resembling that associated with, actual or potential tissue damage.^[1]^ Pain is a subjective and personal experience. It is not only neuronal activity, but also a high-level cognitive process. It is affected by biological, psychological, and social factors to varying degrees.^[1,2]^ Pain is the most common symptom of patients, the most common reason for patients to seek medical treatment, and the most common problem that needs to be dealt with.^[3]^ However, there is still a general lack of appropriate pain management clinically.^[4,5]^ Andersson et al ^[4]^ found that 29% of hospitalized patients reported moderate to severe pain during rest and 41% reported moderate to severe pain during activity. The lack of proper pain management causes the pain problem to persist until after discharge from hospital.^[6,7]^ The pain can even last for more than 3 months and becomes a newly added chronic pain, which affects the patient's quality of life, employment, and sleep.^[4,8]^ It also causes patients to seek medical treatment repeatedly, which indirectly leads to huge increases in costs to the healthcare system.^[9]^ Therefore, providing proper pain management for hospitalized patients is an important issue that cannot be ignored.

To solve the problem of pain control, proper pain assessment is an indispensable first step in pain management. The American Pain Society^[10]^ lists pain assessment as the fifth vital sign to improve the quality of pain management in the entire clinical environment.^[10–12]^ However, merely assessing the degree of pain cannot improve the quality of pain treatment. A complete assessment is needed, especially the patient's psychological distress due to illness or treatment. The patient's emotions include anxiety and depression, which significantly affect the severity of pain. Therefore, multifaceted assessment and management are needed to improve the quality of pain management.^[12]^ Related factors affecting pain control include mood, anxiety, depression,^[2,13]^ gender,^[14]^ age.^[14]^ Vachon-Presseau ^[13]^ stated that people with pain symptoms have a higher incidence of depression than patients without pain. Michaelides and Zis ^[2]^ stated that depression and anxiety in patients will increase their perception of pain. Pain and emotion affect each other and act as risk factors for each other. Fillingim ^[14]^ stated that the factors that affect pain experience include demographic and psychosocial process factors such as age and gender, and the identification of individual factors that impact of pain can provide information for personalized pain management in the future. Therefore, if you can understand the pain control status of inpatients and confirm the factors that affect pain control, it can be used as a reference for clinical care practice and health education, and it will effectively improve the pain control of patients.

Pain control of inpatients is still a problem to be improved. Pain after discharge will affect the quality of life and employment of patients after discharge. Few studies have explored the pain control of inpatients from admission to discharge, and related factors that affect the pain control of patients at discharge. The influencing factors of pain control at discharge from the hospital are still not well understood. With the different home environments after discharge, the patients’ pain control may be affected more. Therefore, this study explores the pain control of inpatients on admission day, hospitalization period, and discharge day, and the factors that affect the pain control of patients at discharge. As a reference for future clinical care practices and health education, it is helpful to effectively improve the pain control of patients and improve the quality of life of patients after discharge.

## 2 Materials and methods

This study was a descriptive and relevant research design, using retrospective medical record data collection of inpatients at a medical center in southern Taiwan from January 2016 to April 2018. Inpatients include patients admitted from an outpatient or emergency department. The inclusion criteria of this study's subjects were: the length of hospital stay ≧ 2 days; age ≧ 20 years old. The exclusion conditions of the study subjects were inpatients in obstetrics, pediatrics, psychiatry, and hospice wards. This study was reviewed and approved by the Institutional Review Board (VGHKS18-CT6-07). Data collected from the medical records of inpatients included age, gender, disease category, admission date, discharge date, hospitalization days, daily pain level, and emotional distress level. The disease category of the patient was classified according to the disease department treated at the time of discharge. The degree of pain and emotional distress obtained in this study were data from patients who self-evaluate the degree of pain and emotional distress during most of the past 24 hours on a daily basis on a 0 to 10 point numerical scale. In this study, data on a total of 73,814 hospitalized patients were collected, and the number of pain and emotional distress person-times was 471,339. Since patients had different numbers of pain and emotional distress data according to the number of days of hospitalization, this study explored the factors affecting the pain degree of discharged patients, taking the pain degree on the discharge day as the dependent variable, so as to avoid the interference of multiple pain data for 1 person.

### 2.1 Statistics

The data was descriptively analyzed by frequency, percentage, average, and standard deviation. The correlation analysis or difference test was used to explore the correlation or difference between variables. A paired sample *t* test was used to explore the difference in average pain scores on admission day, hospitalization period, and discharge day. Multivariate regression analysis was used to explore the predictors of the pain degree of patients on discharge day, and correct the compound influence of each variable. A *P* value of < .05 was considered significant.

## 3 Results

### 3.1 Personal information

There were 73,814 inpatients in this study. The average age of the patients was 59.94 ± 16.49 years old. The average length of stay was 8.11 ± 8.70 days, and the proportion of men was relatively high (54.9%). The proportion of patients by disease category was surgery (54.5%), internal medicine (39.3%), and gynecology (6.2%). The average emotional distress (evaluated from 0 to 10) was 1.32 ± 1.09 at admission, 1.27 ± 0.93 during hospitalization, and 1.07 ± 0.85 on discharge day (Table 1).

**Table 1:** Personal information (N = 73,814 and 471,339 person-times).

Variable	Mean ± SD	n (%)
Age	59.94 ± 16.49	
Hospital days	8.11 ± 8.70	
Sex		
Male		40,521 (54.90)
Female		33,293 (45.10)
Disease category		
Internal medicine		29,005 (39.3)
Surgical		40,234 (54.5)
Gynecology		4575 (6.2)
Emotional distress		
Admission day	1.32 ± 1.09	
During hospitalization	1.27 ± 0.93	
Discharge day	1.07 ± 0.85	

### 3.2 The pain level on admission day, during hospitalization, and discharge day

In this study, there were 73,814 inpatients, and the number of pain assessment records was 471,339. The average pain levels of patients on admission day, hospitalization period, and discharge day were 0.85 ± 1.49, 0.98 ± 1.26, 0.84 ± 1.08, (0–10 numerical scale) respectively, with the highest during the hospitalization period, followed by the admission day and discharge day. The average pain level of the patients during hospitalization was significantly higher than the average pain level on the day of admission (*t* = 28.008, *P* < .001) and the day of discharge (*t* = 34.261, *P* < .001). There was no significant difference between the average pain levels on admission and discharge days. The rates of patients with pain (≥1 point) on admission day, hospitalization period, and discharge day were 31.4%, 48.1%, and 46.5%, respectively. The rate of incidence of pain on discharge day was significantly higher than that on admission day (*t* = 10527.88, *P* < .001). The rate of pain ≥ 4 points on the day of admission, during the hospitalization, and on the day of discharge were 5.8%, 2.7%, and 0.8%, respectively. The rate of pain ≧4 points on admission day was significantly higher than that on discharge day (*t* = 425.07, *P* < .001) (Table 2).

**Table 2:** Pain level on admission day, during hospitalization, and discharge day (N = 73,814 and 471,339 person-times).

	Admission day	During hospitalization	Discharge day	Admission day vs during hospitalization		Admission day vs discharge day		During hospitalization vs discharge day	
Variable	Mean ± SD /n (%)	Mean ± SD /n (%)	Mean ± SD /n (%)	*t* / χ^2^	*P*	*t* / χ^2^	*P*	*t* / χ^2^	*p*
Pain score	0.85 ± 1.49	0.98 ± 1.26	0.84 ± 1.08	−28.008	<.001	1.615	.106	34.261	<.001
Pain				0.063	0.806	10527.88	<.001	0.047	0.830
Yes	23,188 (31.4)	226,880 (48.1)	34,298 (46.5)						
No	50,626 (68.6)	244,459 (51.9)	39,516 (53.5)						
Pain score≧4				3.236	0.072	425.07	<.001	0.038	0.821
Yes	4268 (5.8)	12,884 (2.7)	593 (0.8)						
No	69,546 (94.2)	458,455 (97.3)	73,221 (99.2)						
Pain score									
0	50,626 (68.6)	244,459 (51.86)	39,516 (53.5)						
1	4292 (5.8)	81,627 (17.32)	15,411 (20.9)						
2	7343 (9.9)	78,887 (16.74)	11,054 (15.0)						
3	7285 (9.9)	53,482 (11.35)	7240 (9.8)						
4	1737 (2.4)	6744 (1.43)	380 (0.5)						
5	1489 (2.0)	3901 (0.83)	148 (0.2)						
6	474 (0.6)	1069 (0.23)	26 (0.04)						
7	238 (0.32)	524 (0.11)	20 (0.03)						
8	223 (0.30)	446 (0.09)	14 (0.02)						
9	22 (0.03)	52 (0.01)	1 (0.001)						
10	85 (0.12)	148 (0.03)	4 (0.005)						

### 3.3 Related factors that affect the pain control of patients on discharge day

Table 3 shows the relationship between the patient's age, gender, disease category, hospitalization days, emotional distress, and the patient's pain level on the day of discharge. The factors that were significantly related to the patient's pain on discharge day were age, gender, disease category, length of stay, pain degree on admission day, emotional distress on admission day, and emotional distress on discharge day (*P* < .001). There is a significant negative correlation between age and the patient's pain degree, the higher the age, the lower the pain degree. The degree of pain in men is significantly lower than that in women. The pain degree of surgical patients on discharge day was significantly higher than that of gynecological patients and that of gynecological patients was significantly higher than that of internal medicine patients. The hospital days, the pain level on the admission day, the emotional distress on the admission day, and the emotional distress on the discharge day were significantly positively correlated with the pain level on discharge day. The longer the hospital stay, the higher the degree of pain on the day of admission, the greater the emotional distress on the day of admission, and/or the greater the emotional distress on the day of discharge, then the greater the degree of pain on the day of discharge.

**Table 3:** Related factors of pain control on the patient's discharge day (N = 73,814 and 471,339 person-times).

Variables	Pain score (mean ± SD)	*t*/F/*r*	*P*	Scheffe post hoc test
Age	–	−0.099	<.001	
Sex	–	−19.523	<.001	
Male	0.77 ± 1.05			
Female	0.93 ± 1.10			
Disease department	–	4257.526	<.001	(b)>(c)>(a)
Internal Medicine (a)	0.43 ± 0.84			
Surgery (b)	1.15 ± 1.15			
Gynecology (c)	0.65 ± 0.83			
Hospital days	–	0.018	<.001	
Pain on admission day		0.449	<.001	
Pain during hospitalization		0.002	.648	
Emotional distress on admission day		0.067	<.001	
Emotional distress during hospitalization		0.004	.252	
Emotional distress on discharge day		0.109	<.001	

### 3.4 Multiple regression analysis of the degree of discharge pain

The degree of pain on discharge day was the dependent variable. The independent variables (predictive variables) were disease category (internal medicine, surgery, gynecology), hospitalization days, gender, age, pain degree on admission day, emotional distress on admission day, and the degree of emotional distress on the day of discharge. The data were analyzed by multiple linear regression. After correcting the compound influence of each variable, a total of 7 independent variables that could predict the degree of pain on discharge day were selected as a result. The variance inflation faction of each predicted variable was less than 10 for all 7 variables, indicating that the independent variables had no collinearity problem (Table 4). The explanatory power of the predictive variables of the degree of pain on discharge day was the pain score on the day of admission (0.403), surgery-internal medicine (0.281), discharge day emotional distress (0.135), gynecology-internal medicine (0.052), gender (male-female) (−0.035), age (−0.031), and length of hospital stay (0.022) (Table 4). These results show that the impact on discharge pain is significantly positively correlated with the degree of pain on admission day, emotional score on discharge day, and the hospital stay and that there is a significant negative correlation with age. The degree of pain on the discharge day of men was significantly lower than that of women. Surgery and gynecological patients had significantly higher pain levels on discharge day than internal medicine patients. It means that for every 1 point increase in the patient's pain on admission day, the pain level on discharge day increased by 0.291 points. For every 1 point emotional distress on the discharge day increased, the pain degree on the discharge day increased by 0.171 points. For every additional day of hospitalization, the pain degree on the discharge day increased by 0.003 points. For every 1 year increase in patient age, the pain level on discharge day decreased by 0.002 points. Surgical patients had 0.608 points higher pain on discharge day than medical patients. Gynecological patients had 0.234 points higher pain on the discharge day than internal medicine patients.

**Table 4:** Regression analysis for predicting the degree of discharge pain.

	Unstandardized coefficient		Standardized coefficient			
Model	β estimate	Standard error	Beta distribution	*t*	*P*	VIF
Constant	0.205	0.016		12.739	.000	
Pain on admission day	0.291	0.002	0.403	126.711	.000	1.051
Surgery—internal medicine	0.608	0.007	0.281	82.723	.000	1.196
Emotion score on discharge day	0.171	0.004	0.135	43.225	.000	1.014
Gynecology—internal medicine	0.234	0.015	0.052	15.223	.000	1.231
Gender (male– female)	−0.077	0.007	−0.035	−10.848	.000	1.103
Age	−0.002	0.000	−0.031	−9.680	.000	1.068
Hospital days	0.003	0.000	0.022	6.755	.000	1.054

## 4 Discussion

### 4.1 Differences in pain level between admission day, hospitalization period, and discharge day

The results of this study showed that the rate of pain at admission was 31.4%. The rate of pain degree ≥ 4 points at admission was 5.8%, which was higher than the rate during hospitalization and discharge day, and was significantly higher than the rate on discharge day. It showed that most patients may come to the doctor when the pain was high and uncomfortable. However, the literature rarely mentions the patient's pain status when admitted, and most of the literature mentions the results of a one-time pain survey after hospitalization. The pain level of the patients in this study on the day of admission can be used as reference data.

This study found that the average pain level of patients during hospitalization was significantly higher than the average pain levels on admission and discharge days. The incidence of pain during hospitalization was 48.1%, which was significantly higher than the incidence at admission, indicating that the patient's pain and discomfort increased during the hospitalization. Similar to the study of Ludvigsen et al,^[15]^ the study showed that the incidence of pain in hospitalized patients (numerical scale > 0) was 94% for surgical patients and 70% for medical patients (*P* < 0001). It means that the average pain and incidence of pain may increase due to invasive examination, treatment, and surgery during the patient's hospitalization. It is recommended that patients undergoing invasive examination, treatment or surgery during hospitalization should be appropriately assessed for pain and analgesia. It is necessary to further explore the causes of pain and pain management during hospitalization.

The incidence of pain on the discharge day was significantly higher than that on the admission day. It showed that patients may still have pain when they were discharged from the hospital after undergoing invasive treatment or surgery, and the incidence of pain at discharge is as high as 46.5%. This result is similar to the Veal et al^[7]^ study. That study showed that although most patients had received recommendations on pain self-management, 47.3% of patients still reported moderate to severe pain after discharge, and it is recommended that individualized pain management is needed to optimize patient pain care. A study by Sjøveian and Leegaard ^[6]^ showed that patients hope to obtain more individualized information about pain management before being discharged from the hospital. It is important to give patients pain management advice after discharge, and it is necessary to pay attention to factors that affect personal pain control, and give more individualized management advice.

### 4.2 Predictors of pain control on discharge day pain on admission day

This study found that the degree of pain at admission significantly affected the degree of pain at discharge. This is the same as mentioned in the literature by Swieboda et al ^[16]^ and Wang and Mullally.^[17]^ Swieboda et al^[16]^ stated that individual pain will be affected by their previous pain experience, and that it can even have a profound impact on the individual's pain perception and their behavior and emotional response. Wang and Mullally^[17]^ stated that each person's perception of pain and the degree of pain will be different, depending on their previous experience, cultural background, and situational factors. It is very important to deal with the pain of patients when they are admitted to the hospital, so as to avoid the negative pain perception and emotional response of patients in the future.

### 4.3 Gender

This study showed that women had significantly higher pain scores than men. This is similar to the results of Pieretti et al.^[18]^ Their study found that even with the same symptoms, women feel more pain than men. Packiasabapathy and Sadhasivam ^[19]^ stated that gender is an important factor affecting pain perception, women report more pain than men, women have increased sensitivity to pain, and women show lower pain thresholds and tolerance to painful stimuli. These gender differences are caused by many factors, including the interaction between genetic, physiological, neuronal, hormone, psychological, and social factors.^[18,19]^ Women may also be given the role of caring for family members due to traditional society, so that in addition to the treatment of diseases, they may also worry about the care of children and other members of the family, and the increase in worry will lead to reduced pain tolerance and increased pain, and affect the control of pain.^[2,19]^ It is necessary to further understand the reasons that hinder pain control of female patients and explore the influence of gender on pain management needs.

### 4.4 Disease category

This study found that the disease category will affect the pain level of patients. The average pain level of surgical and gynecology patients is higher than that of internal medicine patients, which may be caused by different perceptions and responses of acute and chronic pain.^[20]^ Because surgical and gynecological patients experience wound pain after surgery, and internal medicine focuses on conservative treatment, the pain of surgical and gynecological patients is higher than that of internal medicine patients. Subramanian et al^[21]^ showed that 97% of patients experienced severe pain after surgery, which interfered with their activities in and out of bed. Over 98% of patients reported feeling severe anxiety due to pain, and 50.5% of people said the pain made them feel helpless. That study's results showed that surgical patients have more pain and more severe pain. Hacker et al^[22]^ stated that gynecological patients will encounter a variety of pain sources, including acute pain secondary to underlying malignant tumors or surgical procedures, and chronic pain associated with malignant tumors and sequelae after treatment. In addition, women have a higher sensitivity to pain,^[19]^ so they have a higher degree of pain than internal medicine patients. Therefore, in the clinical care of patients with pain in different disease categories, attention should be paid to the assessment and management of different acute or chronic pain to reduce pain caused by various sources.

### 4.5 The emotional distress

The study found that the degree of pain on the discharge day of the patient was significantly related to the degree of emotional distress on the day of discharge. As Wang and Mullally ^[17]^ stated, an individual's pain is usually related to anxiety, depression, and disability. Many scholars also stated that pain is a high-level psychosocial cognitive process, which is affected by multiple factors such as biology, psychology, and social factors.^[1,2,23]^ Pain and depression can exacerbate each other.^[24]^ Increased individual anxiety can lead to decreased tolerance to pain and increase the severity of pain.^[2]^ The manifestation of pain is affected by multiple factors. In pain assessment, attention should be paid to the influence of the patient's emotional distress on the patient's pain control to provide appropriate pain management.

### 4.6 Age

This study found that there was a significant negative correlation between age and the pain level of patients, and the elderly had significantly lower pain levels than younger patients. This may be due to the lower sensitivity of the elderly to superficial skin pain.^[14]^ However, the research literature also shows a variety of different results. The study of Wettstein et al^[25]^ showed that age is negatively correlated with pain intensity, but this correlation was not significant. Fillingim^[14]^ indicated that the elderly are less sensitive to transient skin pain but with age, the sensitivity to painful stimuli of deeper tissues increases. It is necessary to further understand the factors that hinder pain control in younger patients to provide appropriate pain management.

### 4.7 Hospital days

This study found that the longer the hospital stays, the higher the pain level on discharge day. As the result of Mayo et al,^[26]^ the high average pain score of hospitalized patients was significantly associated with prolonged hospital stay. These may indicate that it is very important to achieve good pain control during hospitalization and can reduce the number of hospital stays.

## 5 Conclusions

This study found that the incidence of pain on the discharge day of patients was 46.5%, and the incidence of pain on the discharge day was significantly higher than that on admission. This study confirms that the factors predicting the pain of patients on discharge day include the degree of pain on the day of admission, emotional distress at discharge, disease category, gender, age, and length of stay in hospital. Pain is affected by multiple factors, and the understanding of predictors of discharge pain from the results of this study is essential for effective pain assessment and management, which is the basis for individualized pain management. This result can be used as a reference for future clinical care practices, research, and health education. It helps to provide a multilevel assessment of patient pain, give individualized pain management and advice, optimize patient pain management, and improve postdischarge quality of life and activity function of patients.

### 5.1 Limitations on the research

This study did not discuss whether the patients used analgesics, and only compared the pain levels of the patients on the day of admission, during the hospitalization, and the day of discharge. This study did not explore whether patients admitted to the surgical department received surgery. This study did not explore whether there are differences in pain between cancer patients and non-cancer patients. Future research can explore it. The subjects of this study were limited to inpatients in a medical center. Therefore, in general, the results may not be representative of all hospitalized patients. In the future, further research is needed to further clarify the interactions between the biological and psychosocial processes that have an important impact on pain experience. In particular, it is necessary to identify the individual factors and their interactions that lead to the development and persistence of pain.

## Author contributions

LYL conceived and designed the study. LYL contributed to data collection. All authors contributed to data analysis and interpretation. LYL wrote the first draft. All authors critically reviewed the manuscript and approved the version for submission.

**Conceptualization:** Li-Ying Lin, Tzu-Ching Hung, Yen-Ho Lai.

**Data curation:** Li-Ying Lin, Tzu-Ching Hung, Yen-Ho Lai.

**Formal analysis:** Li-Ying Lin.

**Methodology:** Li-Ying Lin.

**Project administration:** Li-Ying Lin.

**Writing – original draft:** Li-Ying Lin.

**Writing – review & editing:** Li-Ying Lin, Tzu-Ching Hung, Yen-Ho Lai.
